# Estrogen Deprivation During Primate ( *Papio anubis* ) Pregnancy: Impact on Systemic Microvascular Flow and Cardiovascular Development and Function After Birth in Offspring

**DOI:** 10.1111/jmp.70076

**Published:** 2026-04-17

**Authors:** Sifa Turan, Jeffery S. Babischkin, Graham W. Aberdeen, Gerald J. Pepe, Eugene D. Albrecht

**Affiliations:** ^1^ Department of Obstetrics, Gynecology and Reproductive Sciences University of Maryland School of Medicine Baltimore Maryland USA; ^2^ Department of Biomedical and Translational Sciences Macon and Joan Brock Virginia Health Sciences at Old Dominion University Norfolk Virginia USA

**Keywords:** aromatase inhibitor letrozole, capillary density, contrast‐enhanced ultrasound (CEUS), ejection fraction, endothelial responsiveness, microbubble replenishment kinetics, skeletal muscle microcirculation, vascular reactivity

## Abstract

**Background:**

Suppressing estradiol (E_2_) during baboon pregnancy lowers offspring skeletal muscle capillary density, vital for insulin‐mediated glucose uptake, and induces insulin resistance. We examined whether E_2_ deprivation also impairs microvascular flow and cardiac function.

**Methods:**

Offspring of untreated baboons, letrozole‐treated baboons, or letrozole plus E_2_ (maternal s.c. injections during the second half of gestation) underwent contrast‐enhanced microbubble ultrasonography to quantify microvessel flow and echocardiography to assess cardiac performance.

**Results:**

Letrozole reduced maternal serum E_2_ by 95% (*p* < 0.01). In letrozole offspring, microbubble flux rate (*β*) fell 55% (*p* < 0.02), replenishment was 5 s slower (*p* < 0.03), and microvessel flow declined 40% (*p* = 0.05); all were restored by added E_2_. Indices of systolic (isovolumic contraction), diastolic (isovolumic relaxation), global performance (Tei index), and cardiac output were unchanged.

**Conclusion:**

Prolonged gestational E_2_ deprivation programs a reduction in microvascular flow without altering cardiac function; maternal E_2_ prevents this, supporting E_2_'s role in optimizing postnatal perfusion and metabolic health.

## 1 Introduction

Central to the Developmental Origins of Health and Disease hypothesis [1], our laboratories have shown that the in utero hormonal *milieu* of human and nonhuman primate pregnancy has an important role in programming mechanisms during fetal development that regulate metabolic function after birth in the offspring. Thus, we have shown that offspring born to baboons administered the aromatase inhibitor letrozole throughout the second half of pregnancy, which suppressed placental estrogen formation, developed insulin resistance and glucose intolerance [2, 3]. These offspring subsequently exhibited compromised first‐phase pancreatic insulin secretion [3], a condition strongly linked to the development of type 2 diabetes mellitus (T2DM) [4].

It is well established that the microvessel unit, comprised of terminal arterioles, capillaries, and venules, is critical for insulin‐regulated glucose uptake in skeletal muscle [5, 6, 7, 8, 9, 10], the predominant site of insulin‐stimulated glucose disposal [11, 12, 13, 14, 15]. Importantly, our recent studies showed that estrogen deprivation during baboon pregnancy reduced fetal and offspring systemic skeletal muscle vascular endothelial growth factor (VEGF) expression [14] and consequently capillary density [15] in offspring born to estrogen‐deprived maternal baboons. It remained to be determined whether these changes in the microvasculature of estrogen‐deprived offspring would alter flow dynamics, potentially exacerbating microvascular function and metabolic function after birth.

In addition to its role in metabolic function, the intrauterine hormonal environment is increasingly recognized as a determinant of cardiovascular health. Cardiovascular disease (CVD) risk has been linked to fetal exposure, highlighting the importance of the prenatal environment [16, 17]. Insulin resistance and cardiovascular dysfunction exhibit a bidirectional relationship, wherein insulin resistance and hyperglycemia contribute to cardiac disease progression, and conversely, cardiac dysfunction fosters insulin resistance [18, 19, 20, 21]. Thus, an unanswered critical question is whether the suppression of placental estrogen production also adversely affects cardiac development and function after birth.

Therefore, the present study tested the hypotheses that placental estrogen suppression throughout the second half of baboon pregnancy impaired systemic microvascular flow as quantified by contrast‐enhanced ultrasonography and cardiac structure and function as measured by echocardiography after birth in the offspring.

## 2 Methods

### 2.1 Animal Treatment Groups

This study was conducted according to the Animal Research: Reporting of In Vivo Experiments (ARRIVE) guidelines. Female baboons ( *Papio anubis* ), originally obtained from the Southwest National Primate Research Center (San Antonio, TX, USA), were individually housed in climate‐controlled rooms with a consistent 12‐h light/dark cycle. All animals were provided standard primate chow (Teklad Primate Diet 2050; Envigo, Frederick, MD, USA), fresh fruit twice daily, and water ad libitum. Female baboons were paired with males for 5 days during midcycle based on menstrual cycle tracking, with pregnancy confirmed via ultrasound.

Animal procedures adhered strictly to the regulations of the U.S. Department of Agriculture and the National Institutes of Health's Guide for the Care and Use of Laboratory Animals (8th edition). The experimental protocol received approval from the Institutional Animal Care and Use Committees at both the University of Maryland School of Medicine and the Macon and Joan Brock Virginia Health Sciences at Old Dominion University. No separate ethical approval certificate number was assigned for this study beyond these institutional IACUC approvals.

Pregnant baboons were randomly assigned to one of three groups:

- Untreated controls: no treatment administered.
- Letrozole treated: Administered daily maternal subcutaneous injection of the aromatase inhibitor letrozole (4,4‐[1,2,3‐triazole‐1‐yl‐methylene]bis‐benzonitrate; Novartis Pharma AG, Basel, Switzerland) at a dosage of 115 μg/kg body weight/day, dissolved in 1.0 mL sesame oil, from gestational day 100 to term (term = 184 days).
- Letrozole plus estradiol treated: Administered daily maternal injections of letrozole (115 μg/kg body weight/day) in combination with progressively increasing doses of estradiol benzoate (initially 25 μg/kg body weight/day starting at day 100, escalating to a maximum of 115 μg/kg/day between gestational day 120 and term) to replicate the physiological rise in estradiol observed in untreated pregnancies.

Between gestational days 100 and 165, maternal blood samples (2 mL) were collected every 5–10 days from a peripheral saphenous vein, following brief restraint and sedation with ketamine HCl (10 mg/kg body weight, intramuscular), for subsequent serum estradiol measurements by radioimmunoassay.

At term, offspring were delivered either spontaneously or by cesarean section and remained with their mothers for 8 months postpartum. Thereafter, offspring were housed in cages adjacent to their mothers, provided standard primate chow (Teklad Primate Diet 2050; Envigo, Frederick, MD, USA), fresh fruit twice daily, and water ad libitum. The present study was comprised of a total of 24 offspring ranging between 2 and 9 years of age ( *Papio anubis* female baboons undergo puberty at 3 1/2 years of age and males at 4 years of age), and the ages of the offspring within the treatment groups in which microvessel flow and cardiac function were performed were similarly distributed. Systemic microvessel flow was assessed under mild anesthesia with 1% isoflurane/nitrous oxide and cardiac function assessed under sedation with intravenous infusion of ketamine/propofol (Ketofol), supplemented with oxygen to maintain appropriate PO_2_ levels. The numbers of offspring in which microvessel flow and cardiac function were conducted within each treatment group are stated in the footnotes of the tables.

### 2.2 Serum Chemistry Analytes

Maternal and offspring serum estradiol levels were measured using an automated chemiluminescent immunoassay system (Immulite; Diagnostic Products Corp., Los Angeles, CA, USA). Serum chemistry analytes (reflecting metabolic, hepatic, and renal function) were quantified by Antech Diagnostics (Lake Success, NY, USA).

### 2.3 Skeletal Muscle Microvessel Flow Quantification by CEU/MB Imaging

Skeletal muscle microvessel flow was quantified using contrast‐enhanced ultrasonography with microbubble imaging (CEU/MB), a validated approach for microvascular quantification [22, 23, 24, 25]. Flow was measured specifically within the forearm musculature, given its predictable microvascular alignment [26]. A lipid‐encapsulated, 2 μm MB contrast agent (Definity, Lumason, North Billerica, MA, USA), prepared in 0.9% saline, was continuously infused (60 mL/h) at a rate of 4.2 × 10^9^ MB/kg body weight/h via an antecubital vein for 10 min.

Ultrasound imaging was performed using an Acuson Sequoia 512 system (Siemens Medical Systems, Mountain View, CA, USA) equipped with a 15L8S transducer set at 7 MHz and applied to the forearm. Following MB collapse induced by increasing the mechanical index from 0.18 to 1.9 for 1 s, MB replenishment in skeletal muscle microvessels was continuously recorded by acoustic signal capture over four to five repeated 60 s intervals (1 frame/300 ms). Axius Auto Tracking Contrast Quantification software was used to generate a time intensity curve of the signal intensity of MB replenishment collected during the 60 s interval and to quantify flow parameters. The data was fit to the program tools and the function *y* = *A* (1 − *e* ^*βt*^). A region of interest (ROI) was drawn on each archived image that excluded the high‐flow larger vessels to confine flow measurement in slower‐filling microvascular beds. From replenishment curves, the following parameters were derived:

- Flux rate (slope; *β*, s^−1^) of and time to attain peak MB replenishment (TTP, s), reflecting microvascular flow velocity.
- Plateau of the video intensity curve (*A*; decibels) and peak intensity (PI; decibels), indicating microvessel blood volume.
- Microvessel flow (*A* × *β*).

### 2.4 Cardiac Function and Structure by 2D Echocardiography, Pulse and Color Doppler Imaging

Cardiac assessments were conducted with a Voluson E8 ultrasound system (GE Healthcare Ultrasound, Milwaukee, WI, USA) as described previously [27].

#### 2.4.1 Cardiac Function

A clear four‐chamber heart view was obtained and pulsed‐wave Doppler gates (1–3 mm) were positioned immediately inferior to the AV valves. Spectral Doppler images capturing three to five consecutive cardiac cycles were obtained to quantify the following parameters:

- Systolic function: Isovolumetric contraction time (ICT), measured from AV valve closure to aortic/pulmonary valve opening.
- Diastolic function: Isovolumetric relaxation time (IRT), measured from closure of aortic/pulmonary valves to AV valve opening, and the early‐to‐late ventricular filling velocity ratios (E/A ratio), indicative of ventricular compliance and atrial contraction effectiveness.
- Global cardiac function: Myocardial performance (Tei) index, calculated as the sum of ICT and IRT divided by ventricular ejection time (ET; duration from aortic valve opening to closure).
- Cardiac output (CO): Calculated as stroke volume (SV, determined by velocity time integral at left ventricular outflow tract multiplied by its cross‐sectional area) multiplied by heart rate (HR).

#### 2.4.2 Cardiac Structure

Cardiac structures were assessed using 2D echocardiography. Detailed cardiac imaging included visualization of the four‐chamber heart, atrioventricular (AV) valves, and the normal crossing of great arteries with an intact interventricular septum. Cardiac biometry was performed by measuring the dimensions of the left and right sides of the heart and the diameters of the great vessels (corrected for body weight). Thicknesses of the aortic valve and the wall of the abdominal aorta and the interventricular septum were measured by M‐mode echocardiography.

### 2.5 Statistical Analysis

Maternal serum estradiol levels and offspring body weights were analyzed by linear regression. Systemic microvascular flow dynamics and components of cardiac function and structure were analyzed by ANOVA or Kruskal–Wallis ANOVA and Tukey–Kramer or Dunn's multiple comparison statistic using GraphPad (San Diego, CA, USA).

## 3 Results

### 3.1 Serum Estradiol Levels

**FIGURE 1:**
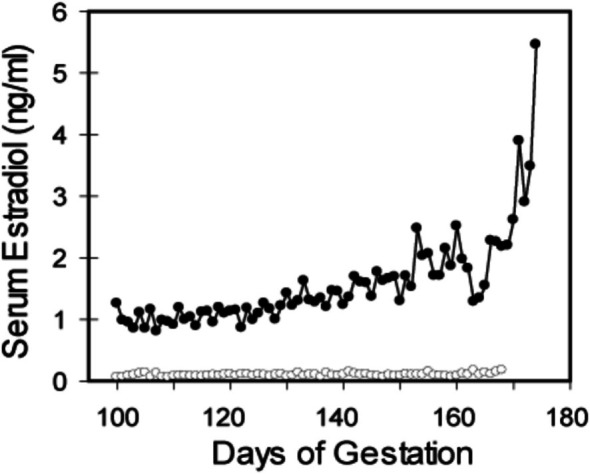
Maternal serum estradiol levels in baboons untreated (●‐●) or treated with letrozole (○‐○) throughout the second half of gestation.

Maternal peripheral serum estradiol levels exhibited a progressive rise during the second half of baboon pregnancy attaining peak levels of over 3 ng/mL near term (Figure 1). The administration of letrozole decreased (*p* < 0.001) maternal serum estradiol levels to less than 0.1 ng/mL throughout the second half of gestation. The administration of letrozole plus estradiol restored maternal serum estradiol to levels that approximated those in untreated baboons (not shown).

As shown previously [14, 15], birth weight, the progressive increase in body weight throughout postnatal life, and serum analyte levels reflecting liver, kidney, and metabolic function were comparable in offspring derived from untreated, letrozole‐treated, and letrozole plus estradiol‐treated baboons. The values for microvessel flow and cardiac function appeared similar in female and male offspring and were not affected by postnatal age of 2–9 years; therefore, values were combined and overall means presented.

### 3.2 Systemic Microvessel Flow Quantification by CEU/MB Imaging

**FIGURE 2:**
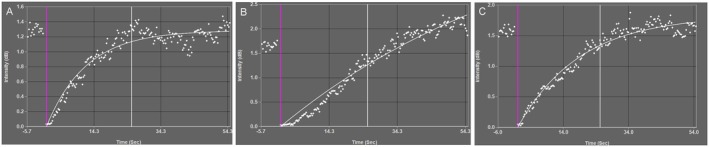
Representative microbubble replenishment kinetic curves assessed by acoustic signal capture in regions of interest (designated by white dots and curved resolution line fit to function *y* = *A* (1 − e^*βt*^)) within forearm skeletal muscle immediately before (time ○, purple vertical line) and during 54 s interval after microbubble burst in offspring derived from untreated (A), letrozole‐treated (B), and letrozole plus estradiol‐treated (C) maternal baboons.

Figure 2 illustrates microbubble (MB) kinetic curves as assessed by CEU acoustic signal capture in regions of interest within forearm skeletal muscle of baboon offspring immediately before (time 0, purple vertical line) and during a 54 s interval of MB replenishment after MB cavitation elicited by increasing the negative acoustic pressure. MB replenishment appeared to occur rapidly (sharp time dependent increase in slope) in offspring of untreated (panel A) and letrozole plus estradiol‐treated (panel C) baboons and slower in offspring delivered to letrozole‐treated baboons (panel B).

**FIGURE 3:**
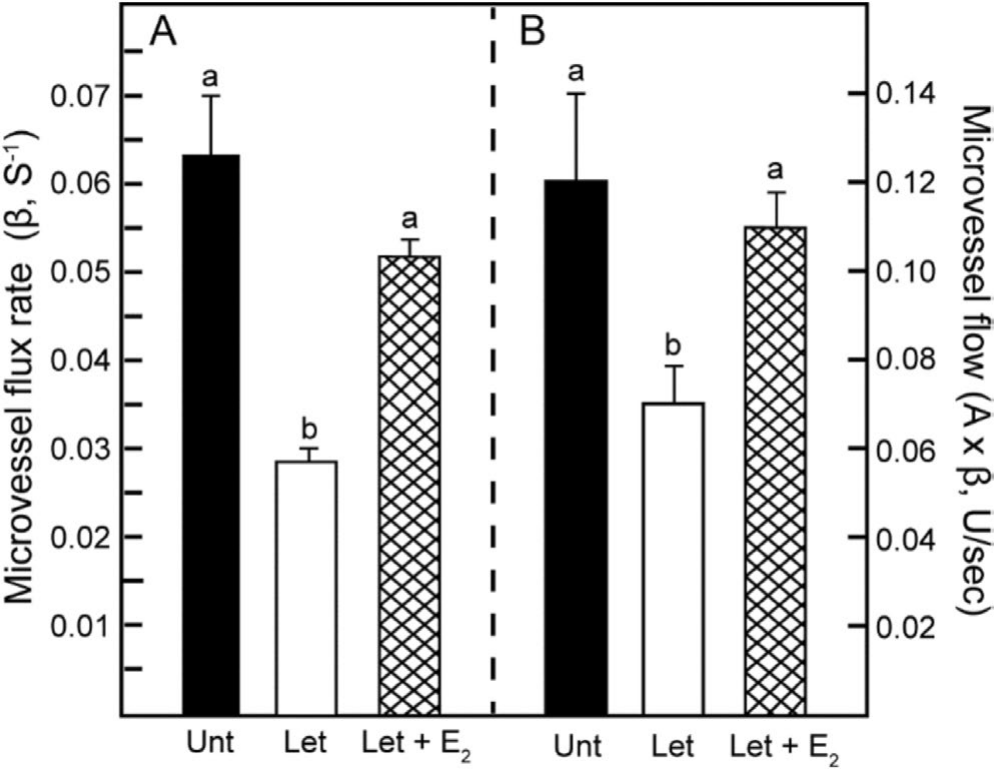
Means ± SE systemic microvessel flow quantified by contrast‐enhanced ultrasonography/microbubble imaging in offspring delivered to maternal baboons untreated (Unt, *n* = 4), treated with letrozole (Let, *n* = 4), or treated with letrozole plus estradiol (Let + E_2_, *n* = 3). Microvessel flux rate is expressed as *β* (rate of microbubble replenishment, panel A) and microvessel flow as A × *β* (blood volume × flux rate, panel B). Values with bars labeled with different letter superscripts are significantly different (*β*, *p* < 0.02; ANOVA, Tukey Kramer test and *A* × *β*, *p* = 0.03–0.05, Kruskal–Wallis ANOVA and Dunn's test).

Consequently, as shown in Table 1 and Figure 3A, the rate (slope, *β*) of MB acoustic signal capture replenishment, reflecting microvessel flux rate, in offspring derived from letrozole‐treated/estrogen suppressed baboons (0.028 ± 0.002) was 55% lower (*p* < 0.02) than in offspring of untreated animals (0.063 ± 0.007) and restored by concomitant letrozole plus estradiol administration (0.052 ± 0.002). Consistent with this change in the rate of MB replenishment, the time required to attain peak MB replenishment (TTP) was approximately 5 s longer (*p* < 0.03) in offspring born to letrozole‐treated (48.15 ± 0.49 s) compared with untreated (43.04 ± 1.73 s) mothers and partially restored by letrozole plus estradiol treatment. However, the plateau of the video intensity curve (*A*) and peak intensity (PI), reflecting microvessel blood volume, were not significantly different in offspring of the three groups (Table 1). Importantly, microvessel flow was 40% lower (*p* = 0.05) in offspring derived from letrozole‐treated/estrogen suppressed mothers (0.07 ± 0.009 U/s) compared with untreated animals (0.12 ± 0.02 U/s) and restored by estradiol administration (0.11 ± 0.008 U/s) (Figure 3B).

**TABLE 1:** Systemic microvessel flow quantified by contrast‐enhanced ultrasonography/microbubble imaging in baboon offspring.

	*β* (s^−1^)	TTP (s)	*A* (U)	Pl (U)
Untreated	0.063 ± 0.007^a^	43.04 ± 1.73^a^	1.85 ± 0.16	1.85 ± 0.21
Letrozole	0.028 ± 0.002^b^	48.15 ± 0.49^b^	2.57 ± 0.22	1.77 ± 0.15
Letrozole + estradiol	0.052 ± 0.002^a^	46.65 ± 0.75^a,b^	2.03 ± 0.22	1.76 ± 0.24

*Note:* Means ± SE skeletal muscle microvessel perfusion as quantified by contrast‐enhanced ultrasonography/microbubble imaging in offspring derived from maternal baboons untreated (*n* = 2 female, *n* = 2 male) or treated with letrozole (*n* = 2 female, *n* = 2 male) or letrozole plus estradiol (*n* = 2 female, *n* = 1 male) on days 100‐term. Values labeled with different letter superscripts are significantly different (*β*, *p* < 0.02; TTP, *p* < 0.03; ANOVA, Tukey Kramer test).

Abbreviations: *β*, rate of microbubble replenishment/flux rate; *A*, plateau of the video intensity curve; PI, peak video intensity; TTP, time to attain peak video intensity.

### 3.3 Cardiac Function and Structure Quantification by 2D Echocardiography, Pulse and Color Doppler Imaging

#### 3.3.1 Cardiac Function

**FIGURE 4:**
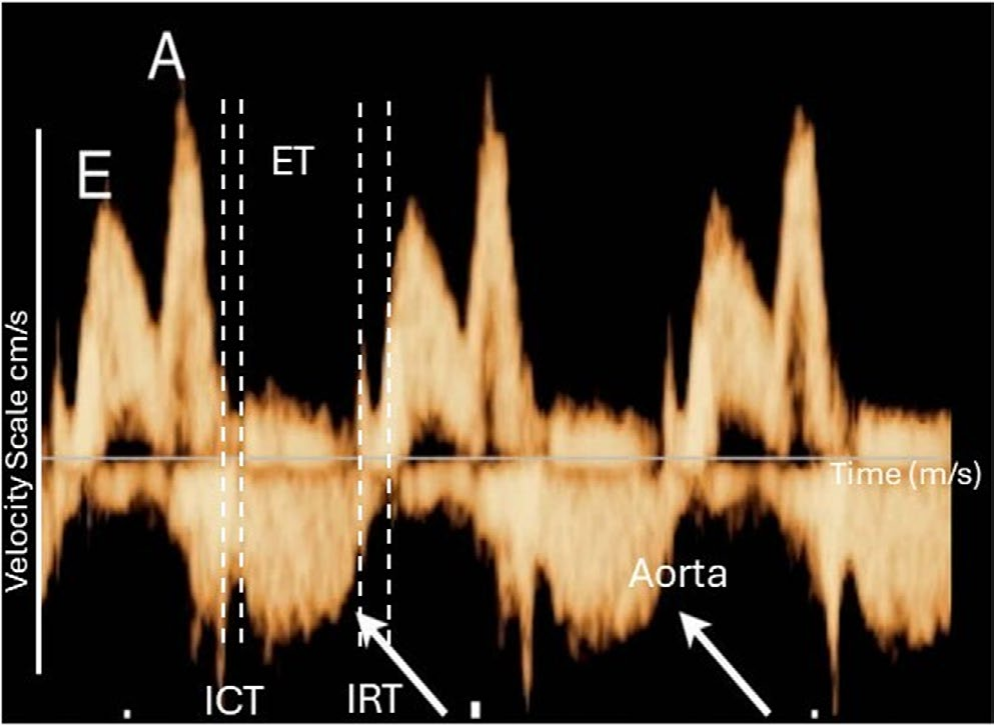
(A) Representative spectral Doppler images of left ventricular inflow and outflow obtained from the hearts of baboon offspring. The inflow Doppler demonstrates the early diastolic (E) and late diastolic (A) velocities across the mitral valve. Arrows indicate the aortic valve Doppler waveform. The interval between mitral valve closure and aortic valve opening represents the isovolumetric contraction time (ICT), the interval between aortic valve opening and closure represents the ejection time (ET), and the interval between aortic valve closure and mitral valve opening represents the isovolumetric relaxation time (IRT).

Figure 4 illustrates representative pulsed Doppler images of ventricular inflow and outflow used to quantify isovolumetric contraction time (ICT), isovolumetric relaxation time (IRT), ejection time (ET), and tricuspid valve early (E) and late (A) wave velocity.

- Systolic function: Isovolumetric contraction time (ICT) was similar across offspring from untreated (0.048 ± 0.003 s), letrozole‐treated (0.049 ± 0.007 s), and letrozole plus estradiol‐treated (0.048 ± 0.007 s) baboons.
- Diastolic function: Isovolumetric relaxation time (IRT) did not differ significantly between offspring of untreated (0.070 ± 0.005 s), letrozole‐treated (0.065 ± 0.007 s), and letrozole plus estradiol‐treated (0.081 ± 0.007 s) mothers. Additionally, early‐to‐late ventricular filling velocity ratios (E/A) measured at the tricuspid valve (TV) and mitral valve (MV) showed no significant differences across groups (Table 2).
- Global cardiac function: The modified myocardial performance index (Tei index) was comparable among offspring of all three treatment groups (Table 2).
- Cardiac output (CO): Cardiac stroke volume (mL/min/kg body weight) and heart rate (beats/min) were similar among offspring from untreated (0.75 ± 0.06 and 95 ± 3, respectively), letrozole‐treated (0.69 ± 0.06 and 90 ± 10), and letrozole plus estradiol‐treated (0.88 ± 0.19 and 87 ± 4) groups. Although cardiac output was approximately 13% lower in the letrozole‐treated group, this difference was not statistically significant (*p* = 0.47, Table 2).

**TABLE 2:** 2D echocardiography, pulse and color Doppler imaging of cardiac structure and function in baboon offspring.

Maternal treatment	TV, E/A	MV, E/A	Tei	CO (L/min/kg)	PA (mm/kg)	Ao (mm/kg)	IVS (mm/kg)
Untreated	1.35 ± 0.10	1.69 ± 0.20	0.525 ± 0.017	0.076 ± 0.006	0.72 ± 0.10	0.69 ± 0.04	5.84 ± 0.78
Letrozole	1.46 ± 0.25	1.49 ± 0.16	0.544 ± 0.062	0.063 ± 0.004	0.52 ± 0.02	0.63 ± 0.03	4.91 ± 0.28
Letrozole + estradiol	1.52 ± 0.25	2.29 ± 0.44	0.602 ± 0.083	0.083 ± 0.015	0.92 ± 0.08	0.88 ± 0.04	7.33 ± 0.89

*Note:* Means (±SE) TV E/A (tricuspid valve early [E] and late [A] wave velocity), MV E/A (mitral valve early and late wave velocity), Tei index (isovolumetric contraction time + isovolumetric relaxation time/ejection time), CO (cardiac output), PA (pulmonary artery diameter), Ao (aortic diameter), and IVS (interventricular septum) thickness in offspring born to untreated (*n* = 4 female, *n* = 4 male), letrozole‐treated (*n* = 2 female, *n* = 3 male) and letrozole plus estradiol‐treated (*n* = 2 female, *n* = 2 male) maternal baboons.

#### 3.3.2 Cardiac Structure

Pulmonary artery diameter, aortic diameter, and interventricular septum thickness were comparable in offspring of the three groups (Table 2). The thicknesses of the aortic valve and wall of the abdominal aorta were similar in offspring derived from untreated (1.9–0.2 mm and 1.1 ± 0.1 mm, respectively), letrozole‐treated (2.7 ± 0.3 mm and 1.1 ± 0.08 mm), and letrozole plus estradiol‐treated (1.9 ± 0.06 mm and 1.5 ± 0.2 mm) baboons.

## 4 Discussion

### 4.1 Main Findings

The present study demonstrated that offspring born to baboons subjected to estrogen deprivation by maternal administration of letrozole throughout the second half of pregnancy exhibited a marked reduction in systemic microvascular flow. Importantly, this decrease was reversed by concomitant maternal administration of letrozole and estradiol. In alignment with our previous findings showing decreased skeletal muscle capillary density in offspring deprived of estrogen in utero [15], the reduction in microvascular flow strongly suggests that placental estrogen production during the second half of primate pregnancy plays a crucial role in programming fetal microvasculature development required for optimal systemic microvessel blood flow after birth.

The microvascular network, composed of terminal arterioles, capillaries, and venules, is fundamental to the delivery of substrates, oxygen, and messengers to target tissues necessary for metabolic function, including insulin‐regulated glucose cell uptake [5, 6, 7, 8, 9, 10]. Consistent with this essential role of the microvasculature on insulin action, the reduction in capillary density and microvessel flow, as shown in the present study, likely accounts for the previously documented insulin resistance and glucose intolerance in offspring born to estrogen‐suppressed baboons [2, 3, 15].

We have previously shown that baboon offspring deprived of estrogen during in utero development exhibited a striking increase in arterial blood pressure after birth [15]. Reductions in microvascular flow and density are established mechanisms contributing to increased peripheral vascular resistance and hypertension [28, 29]. Moreover, there is a complex association between T2DM and hypertension, in which hyperinsulinemia elevates blood pressure via increased vascular resistance and renal sodium reabsorption, whereas hypertension increases the risk of T2DM [30, 31, 32, 33, 34]. Although the present study was not designed to establish the causal relationship between these physiological events, the present and recently completed studies underscore the significance of estrogen during pregnancy in programming mechanisms within the developing fetus that establish vascular and metabolic homeostasis in offspring.

Adverse conditions of human pregnancy, including mutations in the placental CYP19A1 aromatase, steroid sulfatase, and estrogen receptor genes lead to reduced estrogen synthesis or action [35, 36, 37, 38, 39, 40, 41]. Although insulin resistance in offspring has been observed in cases of aromatase mutation during human pregnancy [42, 43, 44], potential effects of estrogen deprivation during human pregnancy on systemic vascular function in the offspring remain unexplored.

Low birthweight preterm babies exhibit a decrease in microvessel density and function and increased incidence of T2DM in adulthood [45, 46, 47]. Low birthweight induced in laboratory animals by placental insufficiency, maternal nutrient restriction or maternal hypoxia also elicits insulin resistance in offspring [48, 49]. However, fetal and offspring growth [15] and uterine blood flow [50] were not changed in letrozole‐treated baboons. Thus, a major advantage of the present experimental approach is the selective reduction of placental estrogen formation to investigate its role in systemic vascular and metabolic function without the confounding effects of reduced placental perfusion and low birth weight.

In contrast to the role that prenatal estrogen plays in programming systemic vascular physiology and metabolic function, cardiac function as measured by echocardiography did not appear to be altered in offspring derived from estrogen deprived baboon pregnancies. Although estrogen has a well‐established role in regulating maternal cardiovascular physiology [51], few studies have been conducted to determine the effect of in utero estrogen on cardiac development and heart function in offspring after birth. Estrogen has been shown to suppress expression of reactive oxygen species within the fetus, thereby protecting female offspring from developing cardiovascular disease [52]. Estrogen also attenuates cardiac hypertrophy in female rodent offspring as elicited by maternal high fat diet treatment [53].

Nathanielsz and coworkers [54, 55, 56] have shown that intrauterine growth restriction induced in baboons by global caloric restriction altered cardiac expression of mitochondrial oxidative phosphorylation subunits and miRNAs of transcription factors in the fetus and reduced systolic and diastolic function in the offspring. Thompson et al. [57] showed that in utero hypoxia in the guinea pig increased the ventricular early/late filling ratio, but had an effect on the Tei index or other aspects of systolic or diastolic function in the fetus at term.

The majority of the studies of the effect of estrogen on cardiac function have been performed in the adult and show a beneficial effect of estrogen on vascular function and cardiac remodeling and performance [58, 59, 60]. Healthy premenopausal women have a lower risk of cardiovascular disease than postmenopausal women and estrogen replacement in postmenopausal women is cardioprotective in restoring ejection fraction and aspects of diastolic and systolic function [59, 61, 62, 63]. Compared with the cardiovascular protective effect of estrogen in the adult, the apparent maintenance of normal heart function in estrogen‐deprived baboon offspring of the current study suggests that cardiac development in the fetus may be resilient or undergo compensatory mechanisms in response to a reduction in estrogen.

### 4.2 Strengths and Limitations

In summary, the present study shows that offspring delivered from maternal baboons deprived of estrogen throughout the second half of gestation displayed a marked decrease in systemic microvessel flow, which was prevented by maternal estradiol administration. However, cardiac function, as measured by echocardiography, was unaltered in offspring from estrogen‐depleted baboon pregnancy. We conclude that the progressive increase in placental estrogen production with advancing primate pregnancy has an important selective role in programming mechanisms within the microvasculature of the developing fetus necessary for optimal systemic microvessel blood flow and metabolic function in the offspring.

A limitation of the present study is that the baboon offspring in which cardiac function was evaluated were relatively young, that is, 2–9 years of age, and therefore it remains unknown whether cardiac dysfunction might emerge in estrogen‐deprived offspring at an older age.

### 4.3 Future Directions

Future investigation incorporating longitudinal imaging, molecular profiling, and functional stress testing may reveal durability and respective mechanisms that underlie the observed changes in estrogen dependent programming of systemic microvascular flow.

## Funding

This work was supported by National Institute of Diabetes and Digestive and Kidney Diseases (R01 DK 120513).

## Conflicts of Interest

The authors declare no conflicts of interest.

## Data Availability

The data that supported the findings of this study are available from the corresponding author on reasonable requests.

## References

[1] D. J. Barker , C. Osmond , J. Golding , D. Kuh , and M. E. Wadsworth , “Growth In Utero, Blood Pressure in Childhood and Adult Life, and Mortality From Cardiovascular Disease,” BMJ (Clinical Research Ed.) 298 (1989): 564–567, 10.1136/bmj.298.6673.564. PMC1835925 2495113

[2] A. Maniu , G. W. Aberdeen , T. J. Lynch , et al., “Estrogen Deprivation in Primate Pregnancy Leads to Insulin Resistance in Offspring,” Journal of Endocrinology 230 (2016): 171–183, 10.1530/JOE-15-0530. 27207093 PMC4946970

[3] G. J. Pepe , A. Maniu , G. Aberdeen , et al., “Insulin Resistance Elicited in Postpubertal Primate Offspring Deprived of Estrogen In Utero,” Endocrine 54 (2016): 788–797, 10.1007/s12020-016-1145-9. 27770396 PMC6038696

[4] S. Del Prato and A. Tiengo , “The Importance of First‐Phase Insulin Secretion: Implications for the Therapy of Type 2 Diabetes Mellitus,” Diabetes/Metabolism Research and Reviews 17 (2001): 164–174, 10.1002/dmrr.198. 11424229

[5] M. G. Clark , E. J. Barrett , M. G. Wallis , M. A. Vincent , and S. Rattigan , “The Microvasculature in Insulin Resistance and Type 2 Diabetes,” Seminars in Vascular Medicine 2 (2002): 21–32, 10.1055/s-2002-23506. 16222593

[6] E. H. Serné , R. T. de Jongh , E. C. Eringa , R. G. IJzerman , and C. D. A. Stehouwer , “Microvascular Dysfunction: A Potential Pathophysiological Role in the Metabolic Syndrome,” Hypertension 50 (2007): 204–211, 10.1161/HYPERTENSIONAHA.107.089680. 17470716

[7] J. D. Chiu , J. M. Richey , L. N. Harrison , et al., “Direct Administration of Insulin Into Skeletal Muscle Reveals That the Transport of Insulin Across the Capillary Endothelium Limits the Time Course of Insulin to Activate Glucose Disposal,” Diabetes 57 (2008): 828–835, 10.2337/db07-1444. 18223011

[8] O. C. Richards , S. M. Raines , and A. D. Attie , “The Role of Blood Vessels, Endothelial Cells, and Vascular Pericytes in Insulin Secretion and Peripheral Insulin Action,” Endocrine Reviews 31 (2010): 343–363, 10.1210/er.2009-0035. 20164242 PMC3365844

[9] T. Akerstrom , L. Laub , K. Vedel , et al., “Increased Skeletal Muscle Capillarization Enhances Insulin Sensitivity,” American Journal of Physiology—Endocrinology and Metabolism 307 (2014): E1105–E1116, 10.1152/ajpendo.00020.2014. 25352432

[10] W. B. Horton and E. J. Barrett , “Microvascular Dysfunction in Diabetes Mellitus and Cardiometabolic Disease,” Endocrine Reviews 42 (2021): 29–55, 10.1210/endrev/bnaa025. 33125468 PMC7846151

[11] R. A. DeFronzo , R. Gunnarsson , O. Björkman , M. Olsson , and J. Wahren , “Effects of Insulin on Peripheral and Splanchnic Glucose Metabolism in Noninsulin‐Dependent (Type II) Diabetes Mellitus,” Journal of Clinical Investigation 76 (1985): 149–155, 10.1172/JCI111938. 3894418 PMC423730

[12] M. A. Abdul‐Ghani and R. A. DeFronzo , “Pathogenesis of Insulin Resistance in Skeletal Muscle,” Journal of Biomedicine and Biotechnology 2010 (2010): 476279, 10.1155/2010/476279. 20445742 PMC2860140

[13] R. R. Wolfe , “The Underappreciated Role of Muscle in Health and Disease,” American Journal of Clinical Nutrition 84 (2006): 475–482, 10.1093/ajcn/84.3.475. 16960159

[14] G. W. Aberdeen , J. S. Babischkin , G. J. Pepe , and E. D. Albrecht , “Estrogen Stimulates Fetal Vascular Endothelial Growth Factor Expression and Microvascularization,” Journal of Endocrinology 262 (2024): e230364, 10.1530/JOE-23-0364. 38738915 PMC11227038

[15] E. D. Albrecht , G. W. Aberdeen , J. S. Babischkin , et al., “Estrogen Promotes Microvascularization in the Fetus and Thus Vascular Function and Insulin Sensitivity in Offspring,” Endocrinology 163 (2022): bqac037, 10.1210/endocr/bqac037. 35325097 PMC9272192

[16] D. J. Barker and S. P. Bagby , “Developmental Antecedents of Cardiovascular Disease: A Historical Perspective,” Journal of the American Society of Nephrology 16 (2005): 2537–2544, 10.1681/ASN.2005020160. 16049070

[17] P. D. Gluckman , W. Cutfield , P. Hofman , and M. A. Hanson , “The Fetal, Neonatal, and Infant Environments—The Long‐Term Consequences for Disease Risk,” Early Human Development 81 (2005): 51–59, 10.1016/j.earlhumdev.2004.10.003. 15707715

[18] S. M. Dunlay , M. M. Givertz , D. Aguilar , et al., “Type 2 Diabetes Mellitus and Heart Failure: A Scientific Statement From the American Heart Association and the American Heart Failure Society of America: This Statement Does Not Represent an Update of the 2017 ACC/AHA/HFSA Heart Failure Guideline Update,” Circulation 140 (2019): e294–e324, 10.1161/CIR.0000000000000691. 31167558

[19] R. Pop‐Busui , J. L. Januzzi , D. Bruemmer , et al., “Heart Failure: An Underappreciated Complication of Diabetes. A Consensus Report of the American Diabetes Association,” Diabetes Care 45 (2022): 1670–1690, 10.2337/dci22-0014. 35796765 PMC9726978

[20] A. A. Oktay , T. K. Paul , C. A. Koch , and C. J. Lavie , “Diabetes, Cardiomyopathy, and Heart Failure,” in Endotext [Internet], ed. K. R. Feingold , S. F. Ahmed , B. Anawalt , et al. (MDText.com Inc., 2000), Last Update: September 26, 2023.

[21] C. Elendu , D. C. Amaechi , T. C. Elendu , et al., “Heart Failure and Diabetes: Understanding the Bidirectional Relationship,” Medicine (Baltimore) 102, no. 37 (2023): e34906, 10.1097/MD.0000000000034906. 37713837 PMC10508577

[22] D. Dawson , M. A. Vincent , E. J. Barrett , et al., “Vascular Recruitment in Skeletal Muscle During Exercise and Hyperinsulinemia Assessed by Contrast Ultrasound,” American Journal of Physiology—Endocrinology and Metabolism 282 (2002): E714–E720, 10.1152/ajpendo.00373.2001. 11832377

[23] M. A. Vincent , D. Dawson , A. D. H. Clark , et al., “Skeletal Muscle Microvascular Recruitment by Physiological Hyperinsulinemia Precedes Increase in Total Blood Flow,” Diabetes 51 (2002): 42–48, 10.2337/diabetes.51.1.42. 11756321

[24] M. Krix , H. Krakowski‐Roosen , H.‐U. Kauczor , S. Delorme , and M.‐A. Weber , “Real‐Time Contrast‐Enhanced Ultrasound for the Assessment of Perfusion Dynamics in Skeletal Muscle,” Ultrasound in Medicine & Biology 35 (2009): 1587–1595, 10.1016/j.ultrasmedbio.2009.05.006. 19682788

[25] P. Rolden , S. Ravi , J. Hodovan , et al., “Myocardial Contrast Echocardiography Assessment of Perfusion Abnormalities in Hypertrophic Cardiomyopathy,” Cardiovascular Ultrasound 20 (2022): 23, 10.1186/s12947-022-00293-2. 36117179 PMC9484161

[26] M. Pascotto , H. Leong‐Poi , B. Kaufmann , et al., “Assessment of Ischemia‐Induced Microvascular Remodeling Using Contrast‐Enhanced Ultrasound Vascular Anatomic Mapping,” Journal of the American Society of Echocardiography 20 (2007): 1100–1108, 10.1016/j.echo.2007.02.016. 17566703

[27] S. Turan , M. R. Asoglu , H. Ozdemir , L. Seger , and O. M. Turan , “Accuracy of the Standardized Early Fetal Heart Assessment in Excluding Major Congenital Heart Defects in High‐Risk Population: A Single‐Center Experience,” Journal of Ultrasound in Medicine 41 (2022): 961–969, 10.1002/jum.15782. 34288033

[28] R. J. Irving , B. R. Walker , J. P. Noon , G. C. M. Watt , D. J. Webb , and A. C. Shore , “Microvascular Correlates of Blood Pressure, Plasma Glucose, and Insulin Resistance in Health,” Cardiovascular Research 53 (2002): 271–276, 10.1016/s0008-6363(01)00450-3. 11744037

[29] D. Rizzoni , C. Agabiti‐Rosei , G. E. M. Boari , M. L. Muiesan , and C. De Ciuceis , “Microcirculation in Hypertension: A Therapeutic Target to Prevent Cardiovascular Disease?,” Journal of Clinical Medicine 12 (2023): 4892, 10.3390/jcm12154892. 37568294 PMC10419740

[30] R. J. Mahler , “Diabetes and Hypertension,” Hormone and Metabolic Research 22 (1990): 599–607, 10.1055/s-2007-1004983. 2076856

[31] G. M. Reaven , “Insulin Resistance, Hyperinsulinemia, Hypertriglyceridemia, and Hypertension. Parallels Between Human Disease and Rodent Models,” Diabetes Care 14 (1991): 195–202, 10.2337/diacare.14.3.195. 2044435

[32] J. R. Petrie , T. J. Guzik , and R. M. Touyz , “Diabetes, Hypertension, and Cardiovascular Disease: Clinical Insights and Vascular Mechanisms,” Canadian Journal of Cardiology 34 (2018): 575–584, 10.1016/j.cjca.2017.12.005. 29459239 PMC5953551

[33] W. D. Strain and P. M. Paldánius , “Diabetes, Cardiovascular Disease and the Microcirculation,” Cardiovascular Diabetology 17 (2018): 57, 10.1186/s12933-018-0703-2. 29669543 PMC5905152

[34] M. A. Hill , Y. Yang , L. Zhang , et al., “Insulin Resistance, Cardiovascular Stiffening and Cardiovascular Disease,” Metabolism 119 (2021): 154766, 10.1016/j.metabol.2021.154766. 33766485

[35] R. Osathanondh , J. Canick , K. J. Ryan , and D. Tulchinsky , “Placental Sulfatase Deficiency: A Case Study,” Journal of Clinical Endocrinology and Metabolism 43 (1976): 208–214, 10.1210/jcem-43-1-208. 133115

[36] C. D. Kashork , V. Reid Sutton , J. S. F. Fonda Allen , et al., “Low or Absent Unconjugated Estriol in Pregnancy: An Indicator for Steroid Sulfatase Deficiency Detectable by Fluorescence *In Situ* Hybridization and Biochemical Analysis,” Prenatal Diagnosis 22 (2022): 1028–1032, 10.1002/pd.466. 12424769

[37] M. H. Herynk and S. A. W. Fuqua , “Estrogen Receptor Mutations in Human Disease,” Endocrine Reviews 25 (2004): 869–898, 10.1210/er.2003-0010. 15583021

[38] B. J. Deroo and K. S. Korach , “Estrogen Receptors and Human Disease,” Journal of Clinical Investigation 116 (2006): 561–570, 10.1172/JCI27987. 16511588 PMC2373424

[39] N. Bouchoucha , D. Samara‐Boustani , A. V. Pandey , et al., “Characterization of a Novel CYP19A1 (Aromatase) R192H Mutation Causing Virilization of a 46,XX Newborn, Undervirilization of the 46,XY Brother, but No Virilization of the Mother During Pregnancies,” Molecular and Cellular Endocrinology 390 (2014): 8–17, 10.1016/j.mce.2014.03.008. 24705274

[40] S. Akçurin , D. Türkkahraman , W.‐Y. Kim , E. Durmaz , J.‐G. Shin , and S.‐J. Lee , “A Novel Null Mutation in P450 Aromatase Gene (CYP19A1) Associated With Development of Hypoplastic Ovaries in Humans,” Journal of Clinical Research in Pediatric Endocrinology 8 (2016): 205–210, 10.4274/jcrpe.2761. 27086564 PMC5096477

[41] V. Bernard , S. Kherra , B. Francou , et al., “Familial Multiplicity of Estrogen Insensitivity Associated With a Loss‐of‐Function *ESR1* Mutation,” Journal of Clinical Endocrinology and Metabolism 102 (2016): 93–99, 10.1210/jc.2016-2749. PMC5413105 27754803

[42] A. Morishima , M. M. Grumbach , E. R. Simpson , C. Fisher , and K. Qin , “Aromatase Deficiency in Male and Female Siblings Caused by a Novel Mutation and the Physiological Role of Estrogens,” Journal of Clinical Endocrinology and Metabolism 80 (1995): 3689–3698, 10.1210/jcem.80.12.8530621. 8530621

[43] K. Takeda , K. Toda , T. Saibara , et al., “Progressive Development of Insulin Resistance Phenotype in Male Mice With Complete Aromatase (CYP19) Deficiency,” Journal of Endocrinology 176 (2003): 237–246, 10.1677/joe.0.1760237. 12553872

[44] G. Libby , D. J. Murphy , N. F. McEwan , et al., “Pre‐Eclampsia and the Later Development of Type 2 Diabetes in Mothers and Their Children: An Intergenerational Study From the Walker Cohort,” Diabetologia 50 (2007): 523–530, 10.1007/s00125-006-0558-z. 17187247

[45] K. L. Goh , A. C. Shore , M. Quinn , and J. E. Tooke , “Impaired Microvascular Vasodilatory Function in 3‐Month‐Old Infants of Low Birth Weight,” Diabetes Care 24 (2001): 1102–1107, 10.2337/diacare.24.6.1102. 11375378

[46] P. L. Hofman , F. Regan , W. E. Jackson , et al., “Premature Birth and Later Insulin Resistance,” New England Journal of Medicine 351 (2004): 2179–2186, 10.1056/NEJMoa042275. 15548778

[47] M. F. Faienza , G. Brunetti , M. Delvecchio , et al., “Vascular Function and Myocardial Performance Indices in Children Born Small for Gestational Age,” Circulation Journal 80 (2016): 958–963, 10.1253/circj.CJ-15-1038. 26861187

[48] S. R. Thorn , T. R. H. Regnault , L. D. Brown , et al., “Intrauterine Growth Restriction Increases Fetal Hepatic Gluconeogenic Capacity and Reduces Messenger Ribonucleic Acid Translation Initiation and Nutrient Sensing in Fetal Liver and Skeletal Muscle,” Endocrinology 150 (2009): 3021–3030, 10.1210/en.2008-1789. 19342452 PMC2703533

[49] J. Choi , C. Li , T. J. McDonald , A. Comuzzie , V. Mattern , and P. W. Nathanielsz , “Emergence of Insulin Resistance in Juvenile Baboon Offspring of Mothers Exposed to Moderate Maternal Nutrient Reduction,” American Journal of Physiology—Regulatory, Integrative and Comparative Physiology 301 (2011): R757–R762, 10.1152/ajpregu.00051.2011. 21653880 PMC3174762

[50] G. W. Aberdeen , A. A. Baschat , C. R. Harman , et al., “Uterine and Fetal Blood Flow Indexes and Fetal Growth Assessment After Chronic Estrogen Suppression in the Second Half of Baboon Pregnancy,” American Journal of Physiology—Heart and Circulatory Physiology 298 (2010): H881–H889, 10.1152/ajpheart.00611.2009. 20023123 PMC2838559

[51] K. L. Thornburg , S. P. Bagby , and G. D. Giraud , “Chapter 43: Maternal Adaptation to Pregnancy,” in Knobil and Neill's Physiology of Reproduction, 4th ed., ed. T. M. Plant and A. J. Zeleznik (Elsevier Academic Press, 2014), 1927–1955.

[52] Z. Chen , L. Wang , J. Ke , and D. Xiao , “Effects of Estrogen in Gender‐Dependent Fetal Programming of Adult Cardiovascular Dysfunction,” Current Vascular Pharmacology 17 (2019): 147–152, 10.2174/1570161116666180301142453. 29493455 PMC6193848

[53] M. Mogi , “Effect of Estrogen on Fetal Programing in Offspring From High‐Fat‐Fed Mothers,” Hypertension Research 45 (2022): 1835–1837, 10.1038/s41440-022-01021-z. 36104624

[54] A. H. Kuo , C. Li , J. Li , H. F. Huber , P. W. Nathanielsz , and G. D. Clarke , “Cardiac Remodelling in a Baboon Model of Intrauterine Growth Restriction Mimics Accelerated Ageing,” Journal of Physiology 595 (2017): 1093–1110, 10.1113/JP272908. 27988927 PMC5309359

[55] S. Muralimanoharan , C. Li , E. S. Nakayasu , et al., “Sexual Dimorphism in the Fetal Cardiac Response to Maternal Nutrient Restriction,” Journal of Molecular and Cellular Cardiology 108 (2017): 181–193, 10.1016/j.yjmcc.2017.06.006. 28641979 PMC5548301

[56] S. P. Pereira , M. S. Diniz , L. C. Tavares , et al., “Characterizing Early Cardiac Metabolic Programming via 30% Maternal Nutrient Reduction During Fetal Development in a Non‐Human Primate Model,” International Journal of Molecular Sciences 24 (2023): 15192, 10.3390/ijms242015192. 37894873 PMC10607248

[57] L. P. Thompson , S. Turan , and G. W. Aberdeen , “Sex Difference and the Effects of Intrauterine Hypoxia on Growth and In Vivo Heart Function of Fetal Guinea Pigs,” American Journal of Physiology—Regulatory, Integrative and Comparative Physiology 319 (2020): R243–R254, 10.1152/ajpregu.00249.2019. 32639864 PMC7509254

[58] V. M. Miller and S. P. Duckles , “Vascular Actions of Estrogens: Functional Implications,” Pharmacological Reviews 60 (2008): 210–241, 10.1124/pr.107.08002. 18579753 PMC2637768

[59] R. A. Khalil , “Potential Approaches to Enhance the Effects of Estrogen on Senescent Blood Vessels and Postmenopausal Cardiovascular Disease,” Cardiovascular & Hematological Agents in Medicinal Chemistry 8 (2010): 29–46, 10.2174/187152510790796156. 20210774 PMC2853974

[60] A. A. Knowlton and D. H. Korzick , “Estrogen and the Female Heart,” Molecular and Cellular Endocrinology 389 (2014): 31–39, 10.1016/j.mce.2014.01.002. 24462775 PMC5709037

[61] A. Iorga , C. M. Cunnigham , S. Moazeni , G. Ruffenbach , S. Umar , and M. Eghbali , “The Protective Role of Estrogen and Estrogen Receptors in Cardiovascular Disease and the Controversial Use of Estrogen Therapy,” Biology of Sex Differences 8 (2017): 33, 10.1186/s13293-017-0152-8. 29065927 PMC5655818

[62] M. A. Lopez‐Pier , Y. Lipovka , M. P. Koppinger , P. R. Harris , and J. P. Konhilas , “The Clinical Impact of Estrogen Loss on Cardiovascular Disease in Menopausal Females,” Medical Research Archives 6 (2018): 1663. 32149188 PMC7059770

[63] D. Xiang , Y. Liu , S. Zhou , E. Zhou , and Y. Wang , “Protective Effects of Estrogen on Cardiovascular Disease Mediated by Oxidative Stress,” Oxidative Medicine and Cellular Longevity 2021 (2021): 5523516, 10.1155/2021/5523516. 34257804 PMC8260319

